# Mechanical Properties of Macro Polypropylene Fibre-Reinforced Concrete

**DOI:** 10.3390/polym13234112

**Published:** 2021-11-25

**Authors:** Rajab Abousnina, Sachindra Premasiri, Vilive Anise, Weena Lokuge, Vanissorn Vimonsatit, Wahid Ferdous, Omar Alajarmeh

**Affiliations:** 1 School of Engineering, Faculty of Science and Engineering, Macquarie University, Macquarie Park, NSW 2109, Australia; rajab.abousnina@mq.edu.au (R.A.); sorn.vimonsatit@mq.edu.au (V.V.); 2 Centre for Future Materials (CFM), University of Southern Queensland, Toowoomba, QLD 4350, Australia; u1108832@umail.usq.edu.au (S.P.); u1106747@umail.usq.edu.au (V.A.); Weena.Lokuge@usq.edu.au (W.L.); Omar.Alajarmeh@usq.edu.au (O.A.); 3 Department of Civil Engineering, Tafila Technical University, Tafila 66110, Jordan

**Keywords:** concrete, macro polypropylene fibre, mechanical properties, CMOD, porosity

## Abstract

Adding fibers to concrete helps enhance its tensile strength and ductility. Synthetic fibres are preferable to steel ones which suffer from corrosion that reduces their functionality with time. More consideration is given to synthetic fibres as they can be sourced from waste plastics and their incorporation in concrete is considered a new recycling pathway. Thus, this work investigates the potential engineering benefits of a pioneering application using extruded macro polyfibres in concrete. Two different fiber dosages, 4 kg/m^3^ and 6 kg/m^3^, were used to investigate their influence based on several physical, mechanical and microstructural tests, including workability, compressive strength, modulus of elasticity, splitting-tensile strength, flexural test, CMOD, pull-out test and porosity. The test results revealed a slight decrease in the workability of the fibre-reinforced concrete, while all the mechanical and microstructural properties were enhanced significantly. It was observed that the compressive, splitting tensile and bonding strength of the concrete with 6 kg/m^3^ fibre dosage increased by 19.4%, 41.9% and 17.8% compared to the plain concrete specimens, respectively. Although there was no impact of the fibres on the modulus of rupture, they significantly increased the toughness, resulting in a progressive type of failure instead of the sudden and brittle type. Moreover, the macroporosity was reduced by the fibre addition, thus increasing the concrete compressive strength. Finally, simplified empirical formulas were developed to predict the mechanical properties of the concrete with fibre addition. The outcome of this study will help to increase the implementation of the recycled plastic waste in concrete mix design and promote a circular economy in the waste industry.

## 1. Introduction

Concrete is the most widely used material for constructing commercial and industrial infrastructure. Despite showing high strength in compression, it has very low tensile strength, which is why it is reinforced with steel bars. The steel reinforcing bars carry the tensile stress and provide more ductility and strength to concrete structures. However, steel reinforcements will eventually corrode and expand, which degrades their functionality and causes microcracks in the concrete affecting the durability of the concrete itself. Corrosion is thus a major concern in the construction industry as in many instances, contractors are unable to maintain the minimum cover required to inhibit further corrosion [1,2]. Moreover, shrinkage cracks can propagate to the steel reinforcement level and accelerate the corrosion in steel bars. Fibre reinforcements such as steel, glass, natural, and synthetic fibres are commonly considered as alternatives to the bar reinforcements [3].

Steel fibres improve the compressive, tensile and flexural strength of concrete due to their high energy absorption and ability to control crack propagation [4,5,6]. However, steel fibres will still corrode, which ultimately leads to a rapid deterioration of concrete structures [7]. On the other hand, glass fibres have excellent strengthening effects but poor resistance to alkali, especially after their inclusion in concrete [8,9], while natural fibres are very cheap and readily available, but they have poor durability and high-water absorption, besides being degradable in nature [10]. Recently, synthetic fibres are receiving more attention from researchers due to the fact they are more environmentally sustainability than other types of fibre [10]. Moreover, synthetic fibres can prevent the formation of the plastic shrinkage cracks in fresh concrete and also improve its post-cracking behaviour [11,12]. Since normal concrete is brittle, concrete structures can only withstand low tensile strain and strength, which is why more ductile synthetic fibres are increasingly being used in general practice. Shah et al. [13] have stated that since concrete is likely to have microcracks during its initial stages, synthetic fibres can be used to improve the quality of normal concrete. In addition, macro- and microsynthetic fibres contribute towards the resistance of macro- and microcracks, respectively.

Kumar and Naik [14] examined how the proportion of synthetic fibres affected the compressive strength of concrete. The 0.05%, 0.1% and 0.15% fibre volume ratios were tested against fibrillation and discretion, and the drying shrinkage of synthetic fibrous concrete and normal concrete were compared [15]. The test results showed a 40% reduction in the drying shrinkage of multi-filament and fibrillated fibrous concrete compared to normal concrete; this reduction is due to the high tensile strength of synthetic fibres contributing to carry more stresses [15]. Furthermore, the drying shrinkage was better controlled when using fibrillated fibre than using multi-filament fibre and therefore fibrillated synthetic fibre is recommended for constructing pavements. Cominoli et al. [16] investigated the possibility of replacing steel mesh in the external plate of a precast panel with synthetic fibres; they found that a minimum cover is no longer needed when fibre reinforced concrete is used, and moreover the panel weight and transportation costs are lower than for a normal precast panel. Further investigation into the post-cracking performance of recycled synthetic fibres in concrete considered the variations between virgin PP fibre and recycled PP fibre under a crack mouth opening displacement [17]. The load at both fibrous concrete cases reached a peak load of 20 kN and then dropped instantly to a range of 0.1 to 0.5 kN; the load then increased until the displacement reached 1.5 mm.

The structural behaviour of concrete containing synthetic fibre under cyclic loading was investigated by Ghosni et al. [18] with the aim of improving the ductility and damping behaviour of concrete beams. The outcome of the study proved that the structure itself could bear the loads without failure when polypropylene (PP) fibre-reinforced concrete was used. Moreover, it was concluded that the use of PP fibres could save the cost of external dampers. Further investigation into the post-cracking performance of steel fibres by Vandewalle [19] consisted of analysing fifteen series of steel fibres with different shapes and sizes in a 3-point bending test. The results indicated that short fibres affected the cracking process at the initial stage and long fibres provide good bonding when there was more deformation [19]. Clarke [20] studied the use of macrofibres in rail infrastructure by replacing traditional concrete slabs with macro synthetic-fibrous concrete panels; macro synthetic fibre concrete slabs have been used in many countries as a replacement for concrete slabs. Macro synthetic-fibrous slabs are faster to manufacture because they do not have any steel reinforcement. A full-scale slab testing was performed to demonstrate the structural performance of macro synthetic fibrous concrete as ground support slabs. In this test, the synthetic fibre volume fraction was 0.32% and 0.48%; these proportions increased the flexural cracking load of concrete by 25% and 32% compared to the normal concrete [21], which are significant values when it comes to building earthquake resistant structures. Moreover, the failure load of the synthetic fibrous concrete was 29% and 44% higher than for normal concrete. The structural reliability of macro polyfibres were further investigated by testing flat slabs reinforced with macro synthetic fibres [22]. It was observed that the tested slabs regained their structural stability by showing considerable ductile behaviour at high loading levels and even during crack propagation. Furthermore, previous studies reported the concrete beams reinforced with 1% of PP fibres in volume improved the ultimate shear strength by 30–45% [23,24]. Moreover, Arslan et al. [25] observed an enhancement in the shear strength by adding PP fibres, however, a sudden drop in load after cracking has been reported. The lesser localised cracking observed on the fibre-reinforced beams compared to the control sample (beams without fibres) indicated that the PP fibres redistributed the shear stresses and improved the bridging action between crack faces before failure. Recently, 0–1.5% volume PP fibres were used for composite railway sleepers [26], and it has been concluded that there was a slight effect on the flow and density of the PFR samples but the porosity gradually increased.

There has been significant progress in the knowledge of using different fibre lengths and fiber ratios for specific physical and mechanical properties. Despite this, none of the previous studies have examined the overall impact of macro polypropylene fibres on concrete properties. Therefore, the study investigated various mechanical. The novelty of the study is to introduce a low-cost fibre system in concrete and to understand how will it affect the mechanical properties. Thus, in this study a comprehensive experimental test considering the use of 4 kg/m^3^ and 6 kg/m^3^ PP fibre dosages added to the control mix were conducted to obtain a better understanding on the effect of PP fibres on physical and mechanical properties (slump test, compression, modulus of elasticity, splitting-tensile test, flexural test, crack mouth opening displacement (CMOD) test, and pull-out test and bond strength. Further investigation was conducted on the effect of PP fibres on the microstructure and porosity. This research will advance the knowledge and help to promote the use of macro poly fibre in some civil engineering applications.

## 2. Materials and Methods

### 2.1. Materials

#### 2.1.1. Fine Aggregate

The methods used to select the aggregate for the experiments in this study are specified in Australian Standard (AS 1726-1994) [27]. The moisture content of the sand was calculated before casting the concrete. The particle size distribution (PSD) of fine sand is based on AS 1141.11.1-2009 [28]. The fine aggregate−grain size of fine aggregate was less than 2.36 mm, and the coarse aggregate was 10 mm crushed gravel.

#### 2.1.2. Macro Polyfibre

**Figure 1:**
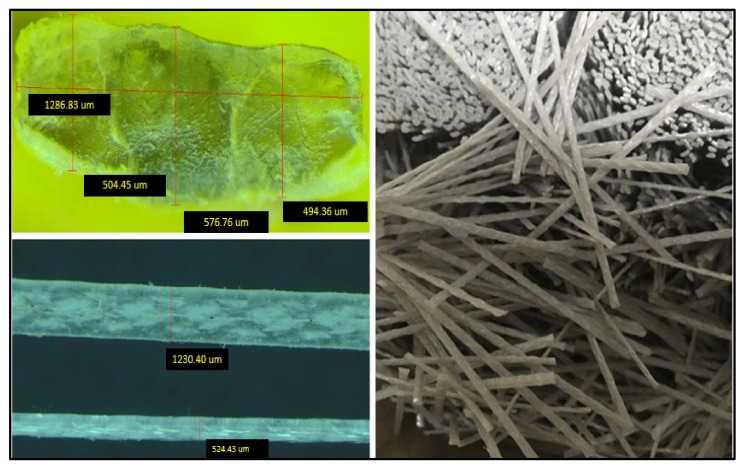
Macro poly fibres.

Macro polyfibres (sourced from FiberconTM, Norwest, NSW, Australia) are a macro synthetic polypropylene fibre (PP) type designed and manufactured in a novel continuously deformed shape to achieve high performance and perfect anchorage to the concrete making them more structurally compliant, as shown in Figure 1. It is worth mentioning that the macro polypropylene fibres are a waste product, which provides environmental as well as cost benefits. In this study, macro polypropylene fibres were used. Table 1 lists the physical and mechanical properties of this type of fibres. The testing methods for metallic fibres are considered to characterise the properties of the MP47 macro poly fibres in accordance to BSEN14651 [29].

### 2.2. Mix Design

The concrete was prepared based on AS 1012.2 [30]; the target compressive strength of the mix was 40 MPa and the ratio of water and cement was 0.5, as per general practice. All the ingredients were mixed together in a 120 L portable electric concrete mixer at a room temperature of 22 °C ± 2. The concrete was cured for 28 days in a fog room with 24 °C and 98% humidity.

### 2.3. Details of the Experiments

Table 2 shows the types of tests and details of the specimens used to study the mechanical properties of the macro polyfibre-reinforced concrete such as slump test, compressive strength and modulus of elasticity, Splitting-tensile strength, flexural test, modulus of rupture, crack mouth opening displacement (CMOD) and bond slip strength. In total, 51 samples were cast to represent different test compressions, and to test the tensile, flexural, bond slip, dog bone, and CMOD of each sample, as shown in Table 2. All the specimens were tested after 28 days of curing.

### 2.4. Test Set-Up and Procedure

#### 2.4.1. Slump Test

**Figure 2:**
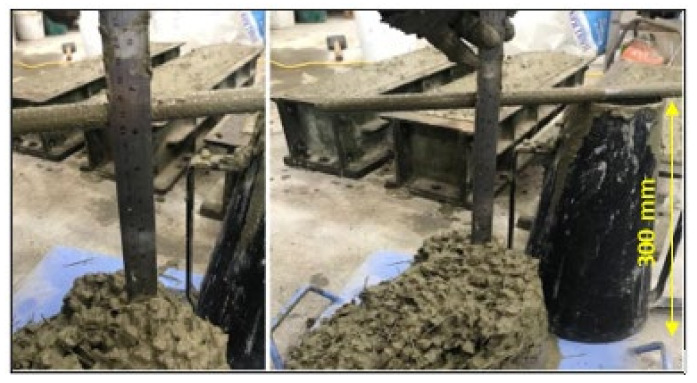
Slump test.

Slump tests of the three batches were carefully performed to ensure the same conditions when measuring the drop of the fresh concrete. All other constituents were kept at the same quantity to obtain accurate slump results. A slump test was carried out at the USQ laboratory according to AS 1012.3.1 as shown in Figure 2.

#### 2.4.2. Compressive and Splitting-Tensile Strength Tests

**Figure 3:**
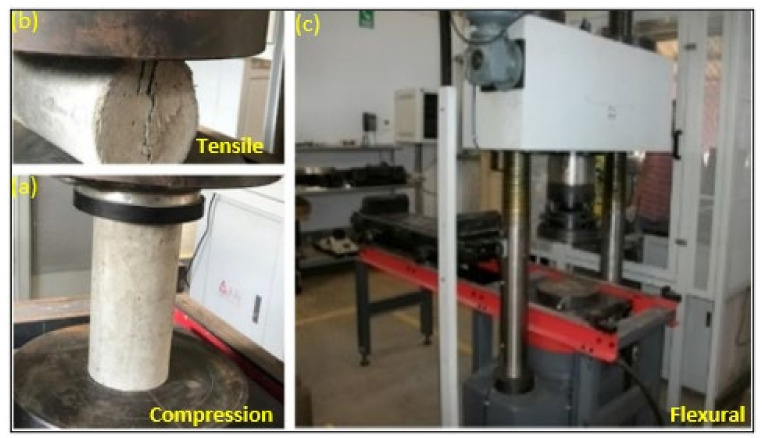
SANS machine (**a**) Tensile, (**b**) Compression, (**c**) Flexural.

Concrete with fibre volume fractions of 1% to 2% is considered ideal (equivalent to nearly 4 kg/m^3^ to 6 kg/m^3^) [31]. Higher doses may increase the strength and stiffness, however, this will cause the workability to reduce and the porosity to increase, affecting durability. A compressive strength test of concrete cylinders with 0 kg/m^3^, 4 kg/m^3^ and 6 kg/m^3^ of macro polyfibre dosages were carried out. These tests followed the procedures prescribed in AS-1012.9 [32]. The specimens were tested to failure using a 2000 kN SANS hydraulic compression and tensile testing machine (Figure 3). The load was applied at a rate of 1.5 mm/min. The maximum load applied to the specimen was then recorded and the type of failure was noted. An average of five samples was used to represent the compressive and tensile strength of the concrete cylinders.

#### 2.4.3. Modulus of Elasticity

The modulus of elasticity test was carried out after 28-days for concrete with different fibre dosages. A compressometer was used to measure the axial shortening of the concrete specimen with the increase of compression load. The readings were taken from the dial gauge for every 10 kN load increment, and the modulus of elasticity was calculated according to AS 1774.31.2 [33].

#### 2.4.4. Flexural Strength Test of Beams

**Figure 4:**
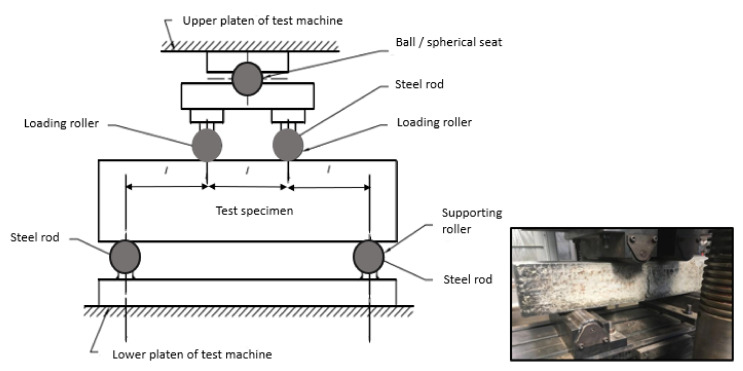
Flexural strength set up.

Flexural tests were conducted using the 500 kN SANS machine shown in Figure 4 and was carried out in accordance to AS 1012.11. The beam dimension was 150 × 150 × 700 mm with a span length of 450 mm. The load rate was 1 mm/min where the data was recorded using the built-in acquisition system built in the SANS machine. Four-point load bending configuration was used to test the beams until failure. In this experiment two beams were prepared and tested from each mix design at 28 days after the casting date. The specimen was placed at the loading base, as shown in Figure 4 and the upper and lower platens were adjusted such that the load can be applied at the centre of the beam. The loads were applied in a uniform pattern, and any cracks or deformation were marked on the beam.

#### 2.4.5. Bond Strength Test

**Figure 5:**
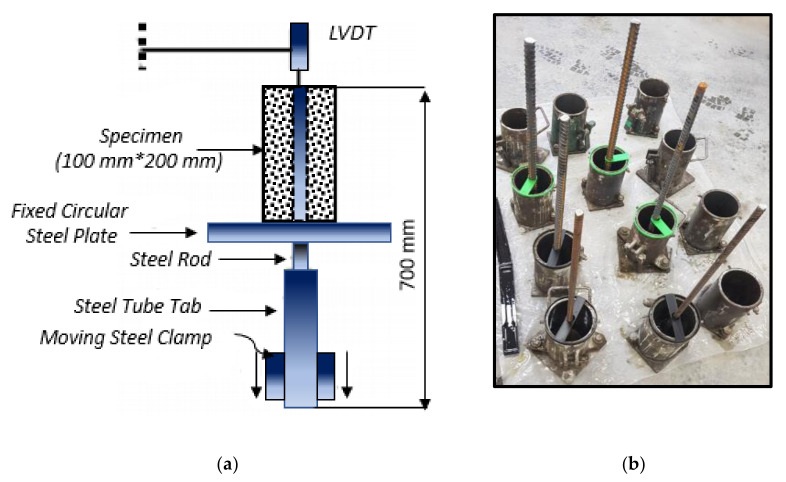
(**a**) Test set up of direct pull-out test and (**b**) the plastic braces.

Figure 5a shows the schematic diagram and set up of the direct pull-out test used in this study. This test was conducted in accordance with ACI 440.03R-04 [34]. The concrete specimens were 100 mm diameter and 200 mm height with steel bars embedded along the centre having various diameters of 12, 16 and 20 mm. In order to locate the bars at the centre of the moulds, plastic braces (Figure 5b) were created at the USQ 3D-Makerspace Laboratory. The specimen was positioned upside down while the bar was pulled down at a constant rate of 1 mm/min. A single linear variable differential transducer (LVDT) was placed at the end of the steel bar to measure the overall slip displacement relative to the fixed concrete specimen. A support stand for the LVDT was placed separately from the test specimen to ensure that any movement or failure during the loading stage would not affect the measurements. The pull-out load and end-slip were measured and recorded using system 5000 data logger.

#### 2.4.6. Crack Mouth Opening Displacement (CMOD)

**Figure 6:**
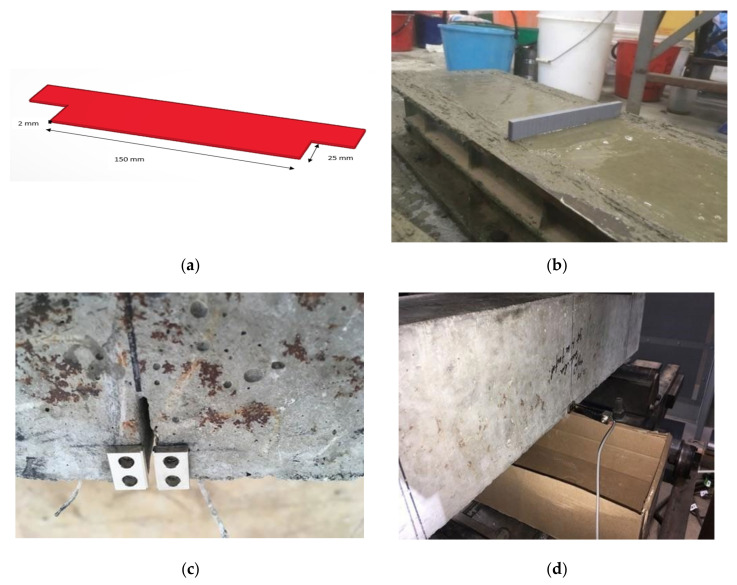
Experimental test procedure of the CMOD. (**a**) 3D printed plastic strip. (**b**) Placement of the plastic strip. (**c**) Steel clips. (**d**) Attached transducer.

The concrete beams reinforced with and without fibres have been tested to investigate load–deflection as well as crack mouth opening displacement (CMOD) behaviour. This test has been conducted to determine the effect of the fibre addition on the crack initiation and propagation as well as the post-cracking behaviour. A notch placed at the bottom part of the beam was the main difference between conventional flexural test and the CMOD test to accurately capture the first and main crack. To create the notch, a 3D printed plastic strip (see Figure 6a) with 2 mm width and 25 mm depth was made and placed onto the fresh-cast concrete beam, as shown in Figure 6b. The test samples were prepared and tested as per BS EN 14651-2005 [29]. The notch was cleaned, and two steel clips were glued at the bottom of either side of the notch (Figure 6c,d). Afterwards, a transducer with UB-5A model was attached to the steel clips to measure the opening displacement of the crack. The specifications of the transducer are listed in Table 3. The CMOD data was recorded by system 5000 data logger.

#### 2.4.7. Void Measurement and Microscopic Observations

**Figure 7:**
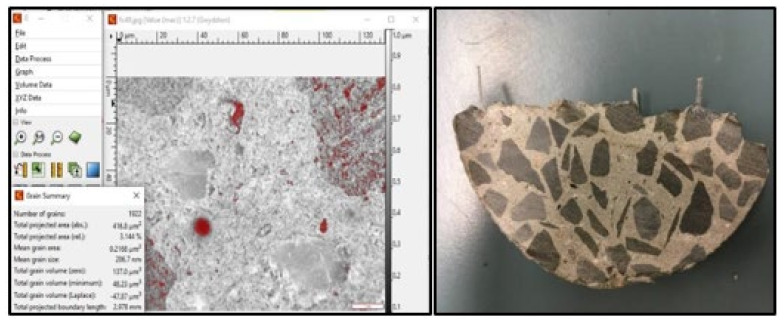
Example of image analysis using Gwyddion software.

A microscope (SMZ-168 series, Motic, Hong Kong) was used to examine the microstructure and measure the pores diameter at the internal fracture surface of the concrete cylinders. Moreover, the porosity of tested specimens was analysed using the Gwyddion software (Figure 7).

## 3. Results and Discussion

### 3.1. Slump Test

**Figure 8:**
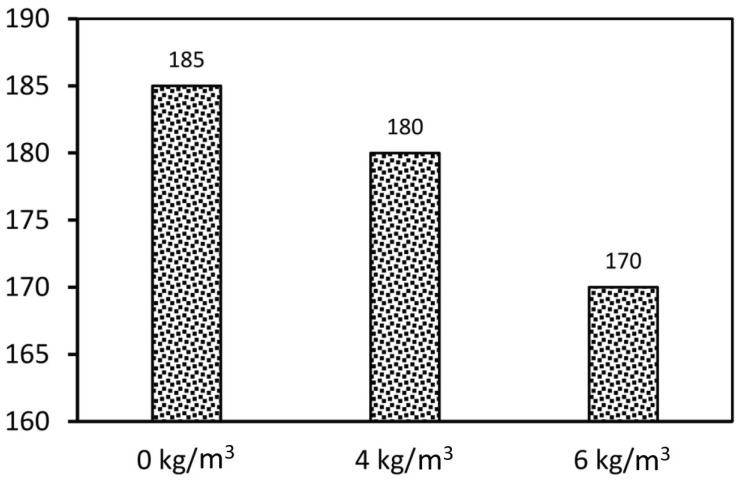
Slump test results of concrete with different fibre content.

Figure 8 shows the slump of the fiber-reinforced concrete. The results showed that the slump value of the tested specimens varied between 170 and 185 mm. The slump changed due to the increasing the fiber content and form. For instance, the slump of 4 kg/m^3^ and 6 kg/m^3^ concrete dropped by 2.7% and 8.1% respectively compared to the normal mix of concrete with 0 kg/m^3^ of fibre. The specimen with 6 kg/m^3^ recorded a reduction in workability by 8.1% compared to the specimen without fibres. Due to larger surface area, fibers need to adsorb a lot of cement paste to wrap around them, which increases the viscosity of the mixture. Fibres form a network structure in the concrete which is restraining the fresh concrete from flow [35,36,37].

### 3.2. Compressive Behaviour

#### 3.2.1. Failure Mechanisms

**Figure 9:**
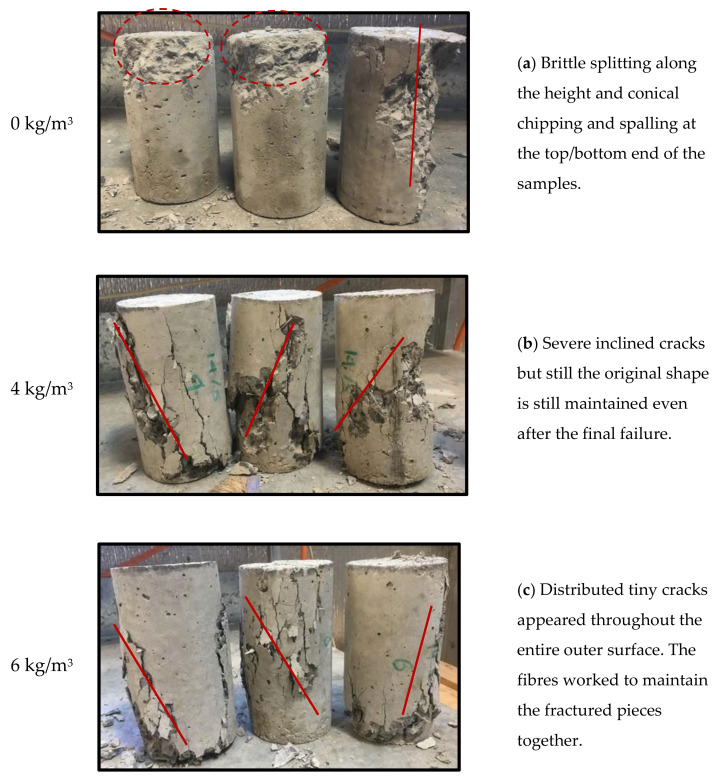
Failure modes of the tested specimens with different fibre dosage. (**a**) Splitting and conical fracture. (**b**) Shear failure. (**c**) Shear failure.

Different failure mechanisms of the fibre-reinforced concrete under compression were observed. Figure 9 shows the modes of failure of the tested cylinders with and without fibres. It can be noticed that the control samples with 0 kg/m^3^ shows splitting cracks along the height and localised crushing at the top/bottom ends of the cylinders (Figure 9a). This type of failure is reported as a possible failure mode for plain concrete, which is normally caused by the friction angle of the concrete (the sample with vertical splitting) and the friction between the loading steel plate and the sample surface (for the samples failed at the end portion) [38,39,40]. The inclusion of the macro polyfibres in the concrete considerably changed the mode of failure to shear failure (Figure 9b,c). This can be due to the additional bonding and tensile resistance by the fibres which changed the progression of the vertical cracks into inclined shear ones. It is worth mentioning that increasing the fibre dosage from 4 kg/m^3^ to 6 kg/m^3^ mitigated the severity of the shear cracks and make the crack more distributed which is due to the increase in the bond between the concrete parts allowing more uniform distribution of the tensile stresses internally evidenced by the intact crushed parts of the fibrous concrete even after the final failure. Similar failure observations previously [28,33,34,38] were addressed by adding fibres to concrete specimens and this modified their failure by enhancing the mechanical properties, thus reducing the risk of catastrophic failure.

#### 3.2.2. Compressive Strength and Stress-Strain Behaviour ($\sigma_{c}$)

**Figure 10:**
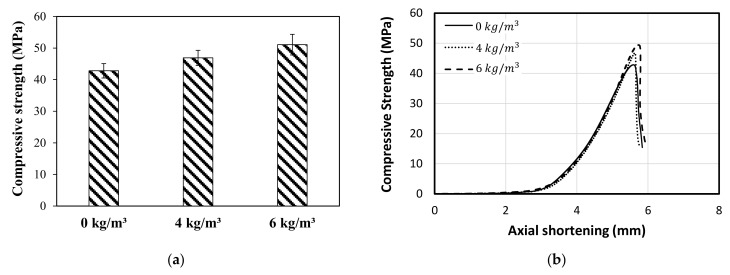
(**a**) Average compressive strength vs. fibre content, and (**b**) Stress-displacement behavior of tested cylinders with various dosage of macro poly fibres.

The compressive strength ($\sigma_{c}$) of concrete was calculated by dividing the maximum failure load by the actual cross-sectional area of each sample. Table 4 shows the compressive strength (MPa) results of concrete with 0 kg/m^3^, 4 kg/m^3^, and 6 kg/m^3^ of fibre dosage. According to Table 4, moreover, the tested cylinders revealed low standard deviation and coefficient of variation values, which indicates higher consistency, good mixing and casting procedures. Figure 10a shows a gradual increase in the $\sigma_{c}$ with the fibre addition, thus, it can be observed that adding 4 kg/m^3^ and 6 kg/m^3^ dosage of fibres increased the $\sigma_{c}$ by 9.6% and 19.4%, respectively. Figure 10b, moreover, presents the compressive behaviour of the tested cylinders against the axial shortening where they showed insignificant effect of the fibre addition on the axial stiffness even though the plastic fibres are less stiff than concrete. This might be due to the low volume occupation of the fibres compared to the volume of the cylinder which is less than 0.7% [31]. Generally, the elastic modulus of the fibres contributes negatively or positively to the concrete compressive strength. Nevertheless, the length, distribution and the surface finish of the fibres are also considered to impact the compressive strength. In this study, it was clear in Figure 8 how the fibres changed the mode of failure and the failure mechanism of the concrete cylinders with different amounts of fibre dosage which indicated new stress pathways inside the cylinders after adding the fibres. Indeed, the role of fibres is to resist the transverse tensile and shear stresses resulting in more uniformity in the stress distribution leading to higher axial load resistance as a result. These findings are consistent with the reported results by Hasan et al. [41], who observed 7% increase in the compressive strength of concrete when adding 0.5% by weight of PP fibres. Moreover, they have noticed that the failure cracks are changed from longitudinal to inclined direction. Nevertheless, Choi and Yuan noticed no significant difference in the compressive strength between the sample with and without fibres. This might be attributed to the shorter and thinner fibres they have used (19 mm in length and 0.013 in diameter) as observed by Li et al. [42].

#### 3.2.3. Modulus of Elasticity

**Figure 11:**
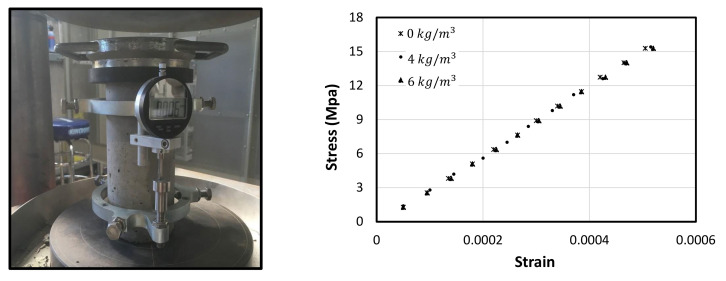
Stress-strain relationship within the elastic limit.

The effect of macro polyfibre on the modulus of elasticity has been investigated, which is given by the slope of the stress-strain curve within the proportional limit of the concrete, as shown in Figure 11. The modulus of elasticity of the concrete with no fibres was 31.0 GPa., while a brief decrease in the modulus of elasticity by 1.6% (30.5) and 2.0% (30.4) was noticed when adding 4 kg/m^3^ and 6 kg/m^3^ fibre dosage to the concrete, respectively. This is attributed to the fact that the elastic modulus of the plastic fibre (around 900 kg/m^3^) is lower than that of concrete (around 2500 kg/m^3^) which negatively affects the total modulus of elasticity. Nevertheless, the volume of the added fibres was less than 0.7% at maximum, which showed insignificant reduction to the modulus of elasticity value as evidenced by the unchanged in the axial compression stiffness in Figure 10b.

#### 3.2.4. Splitting-Tensile Strength

**Figure 12:**
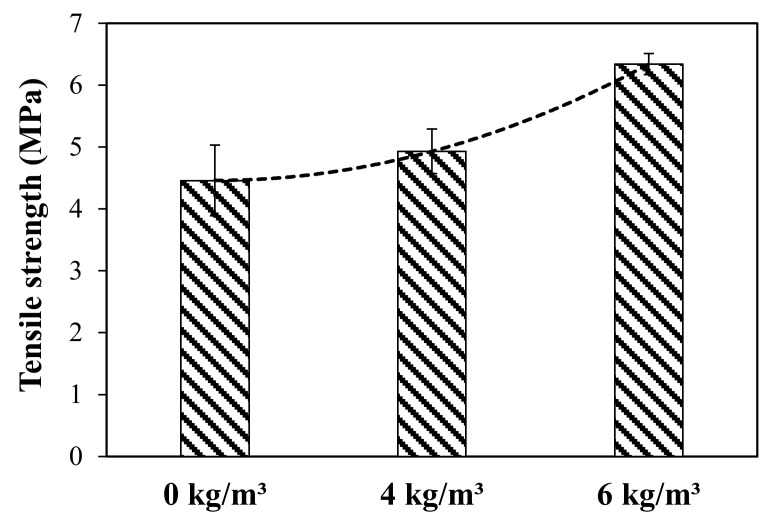
Average splitting-tensile strength vs. fibre dosage.

The impact of macro polyfibres on the splitting-tensile strength has been investigated in this study. The test results of the splitting-tensile strength ($\sigma_{ts}$) (MPa) of concrete specimens with 0 kg/m^3^, 4 kg/m^3^, and 6 kg/m^3^ of fibre dosage are presented in Table 5 and Figure 12. The $\sigma_{ts}$ is calculated using Equation (1), where P, L and D are the maximum load, length of the specimen (200 mm) and the diameter of the specimen (100 mm), respectively. As predicted, the samples with 6 kg/m^3^ of fibre showed the highest $\sigma_{ts}$ values with an average of 6.34 MPa, which is 28.7% and 41.9% higher than the samples with 4 kg/m^3^ and 0 kg/m^3^, respectively. This is due to the role of the PP fibres to uniformly distribute the internal stresses preventing the initiation of the cracks and reinforcing them once initiated against propagation. This led to an increase in the tensile resistance of the concrete. The results show that the increase in the fibre content from 4 kg/m^3^ to 6 kg/m^3^ achieved a higher increase rate of $\sigma_{ts}$ value than the increase from 0 kg/m^3^ to 4 kg/m^3^. This could be due to the increase of the volume occupation by adding 6 kg/m^3^ fibre dosage which reinforced more volume in the tri-axial space of the tested specimens leading to increase the $\sigma_{ts}$ rate improvement. Similar findings can be found by Hasan et al. [41] and Song et al. [43], who concluded that the improvement in the flexural and splitting tensile strength is not linearly proportional to the addition of the PP fibres where the increase in the PP fibre dosage exponentially increased the flexural and splitting tensile strength. Moreover, high consistency in the $\sigma_{ts}$ values can be observed in the fibrous concrete samples compared to the control samples. This is evidenced by the low standard deviation and coefficient of variance values, as reported in Table 5.

Splitting-tensile strength:

(1) $$\sigma_{ts}=\frac{2P}{\pi LD}$$

### 3.3. Flexural Behaviour

#### 3.3.1. Mode of Failure

**Figure 13:**
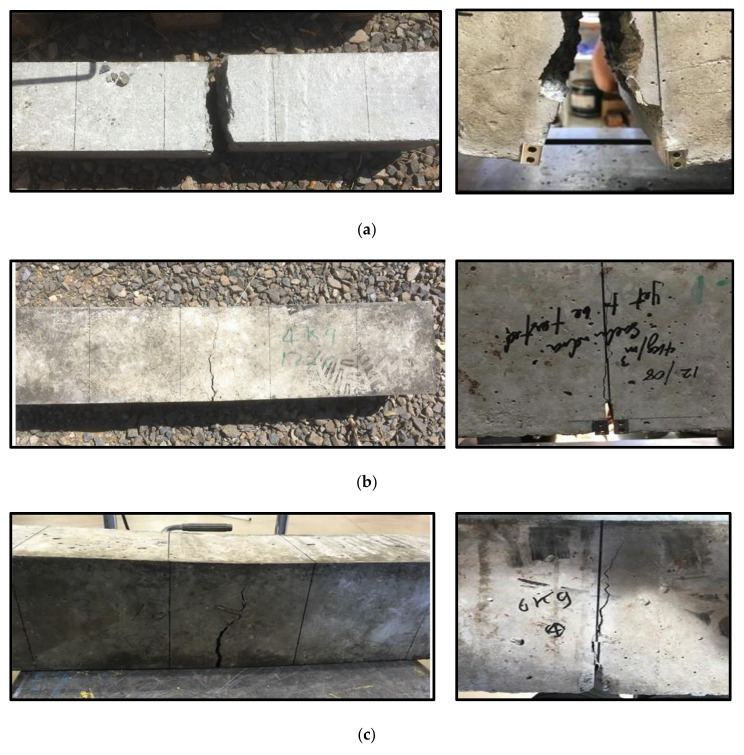
Final failure of the tested beams (left hand side) and CMOD (right hand side). (**a**) 0 kg/m^3^ fibre mix. (**b**) 4 kg/m^3^ fibre mix. (**c**) 6 kg/m^3^ fibre mix.

Figure 13 shows the final failure of the concrete beams with different fibre dosage. Due to the fact that the plain concrete beams split into two pieces in a brittle manner, the beams with 0 kg/m^3^ showed similar behaviour after reaching the modulus of rupture (see Figure 13a), which refers to the localised tensile stress along the height of the beam. In contrast, the concrete beams with 4 kg/m^3^ fibre dosage showed a tiny crack at the middle length of the beam where the crack mouth opening was attached at that moment. The observed crack propagated until it reached almost the top of the beam where the final failure occurred, as observed in Figure 13b. During the test some snipping sound was heard which might be due to breaking in the fibres. Similar observation was noticed for the beams reinforced with 6 kg/m^3^ macro polyfibres, as shown in Figure 13c. Both fibrous concrete beams have shown progressive failure without separation even after the final failure. This was due to the presence of the macro polyfibres which resisted and distributed the tensile forces caused by bending and mitigated the crack propagation.

#### 3.3.2. Flexural Strength

**Figure 14:**
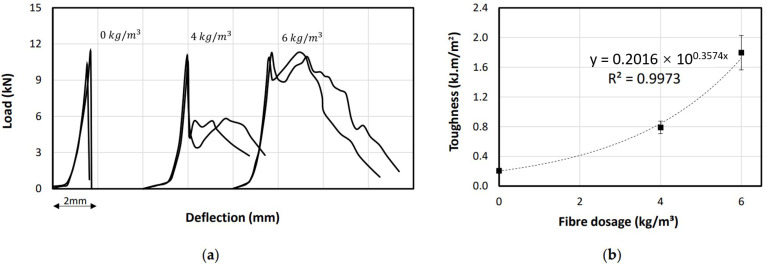
Flexural behaviour of the tested beams. (**a**) Load-deflection behavior. (**b**) Toughness behavior.

All the concrete beams reinforced with and without macro PP fibres were tested until failure to determine the influence of the fibres on the flexural behaviour including peak flexural strength ($\sigma_{f}$), maximum deflection, and the post-peak behaviour. Test results are reported in Table 6, the flexural strength (modulus of rupture) of the beams was calculated using Equation (2). It can be observed that the fibres have an insignificant influence on the flexural strength of the tested beams even with the inclusion of the PP fibres. Figure 14a shows the typical load-deflection behaviour of the tested beams. All the tested beams have almost similar peak load and deformation at the peak load, which indicates that the fibre content does not affect the flexural capacity. The initial crack and the crack propagation until reaching the peak load, especially for flexural members as they are dominated directly by the plain concrete properties [30]. Nevertheless, the post-peak behaviour of the tested beams was significantly vary based on the fibre dosage addition. Therefore, once the crack appears the flexural stresses transmit to the PP fibres which make the failure more progressive and delay the final failure by blocking the pathway progression of the cracks and resist the tensile forces at the bottom portion of the beam. This is called ductile behaviour according to previous studies [44,45] at which the PP fibres prevent the sudden brittle failure of the plain concrete. In Figure 14a, the plain concrete beams showed a brittle failure with the sudden drop of the load leading to splitting the beam into two pieces. The beam with 4 kg/m^3^ fibre dosage showed a 60–70% drop in the peak load due to the first crack appearance. Afterwards, the PP fibres maintained the load resistance up to almost 50% of the peak load. The load resistance gradually dropped again indicating the progression of existing crack reaching the topmost height of the beam leading to the final failure. Similarly, the beam with 6 kg/m^3^ fibre dosage showed a drop in the load resistance after the peak but only 20% due to the higher fibre dosage compared to the one with 4 kg/m^3^. Interestingly, the full load resistance was maintained because of the PP fibres to the point the load resistance decreased due to the crack propagation to the topmost height of the beam. It is worth mentioning that the load increase in the beams with fibres (4 kg/m^3^ and 6 kg/m^3^) is due to the high strength PP fibres used in this study (550 MPa), while the ordinary behaviour of the PP fibre-reinforced beams is different by showing gradual decrease in the load resistance until the final failure. Fibres have the ability to resist shear across cracks, an enhanced residual strength, and enhanced energy dissipation [46].

The post-peak behaviour is a very important reliability-aspect of the concrete structures at which it indicates the energy absorption capacity (toughness) of a structure by showing strain hardening behaviour [47]. This is considered an extra safety factor for a structure which might be subjected to a sudden load exceeding the peak strength capacity. In this study, the flexural toughness value (T) was calculated by the area under the load-deflection curve divided by the cross-sectional area (A), see Equation (3). Figure 14b shows that the beams with 6 kg/m^3^ fibre dosage had 228% and 857% increase in the T value compared to the beams with 4 kg/m^3^ and 0 kg/m^3^ fibre dosage, respectively. It was clearly noticeable that flexural toughness value of the concrete beams was proportion to the PP fibre dosage.

In Equations (2) and (3), $P\left(x\right)$, $P_{max}$, δ, A, l, B and D are the load corresponding to a certain defection, maximum peak load, maximum deflection, cross-sectional area (150 mm × 150 mm), span length (150 mm), beam width (150 mm) and beam height (150 mm).

Flexural strength (modulus of rupture):

(2) $$\sigma_{f}=\frac{MC}{I}=\frac{P_{max}\;l}{B\;D^{2}}$$

Energy absorption (toughness):

(3) $$T=\frac{\int_{0}^{\delta }P\left(x\right).dx}{A}$$

#### 3.3.3. Crack Mouth Opening Displacement (CMOD)

**Figure 15:**
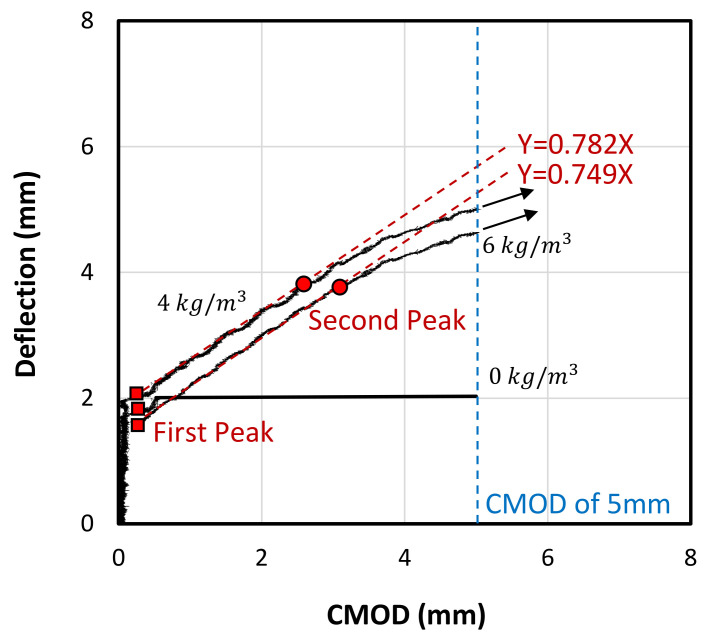
CMOD vs. deflection of the tested beams with different fibre dosage.

Figure 15 shows the relationship between CMOD and the deflection of the concrete beams with 0 kg/m^3^, 4 kg/m^3^ and 6 kg/m^3^ of macro polyfibre. It can be seen that the concrete beam without fibres showed no cracking until reaching around 2 mm deflection. Then a crack appeared and suddenly propagated to the topmost height of the beam leading to split the beam into two pieces. On the other hand, the beams with 4 kg/m^3^ and 6 kg/m^3^ of fibre dosage showed crack initiation almost at the same deflection (2 mm) (same observation was noticed in Figure 14a). In contrast to the plain concrete beam, the fibrous concrete beams revealed a linear increase in the CMOD in relation with the beam deflection until reaching almost 4 mm. This behaviour represents the zone between the load drop and the second peak load in Figure 14a. Afterwards, the relationship between the CMOD and deflection became nonlinear where the concrete started showing gradual decrease in the load resistance in Figure 14a. This might be due to the well-known non-linear behaviour of the concrete under compression in the top portion of the beam. It should be mentioned that the CMOD recording stopped at 5 mm as it reached its maximum capacity. It was found that the slope of the linear ascending lines between CMOD and deflection for beams with 4 kg/m^3^ and 6 kg/m^3^ of fibre dosage was very close. Thus, a relationship between the CMOD and deflection was generated, as can be seen in Equation (4), where the slope value in this equation was the average of the two slope values of both lines. Interestingly, the slope value (0.765) in Equation (4), proposed by Ding [48] for steel fibre-reinforced beams, falls in the range (0.65 to 1.12):

(4) Deflection=0.765CMOD

### 3.4. Bonding and Pull-Out Behaviour

#### 3.4.1. Mode of Failure

**Figure 16:**
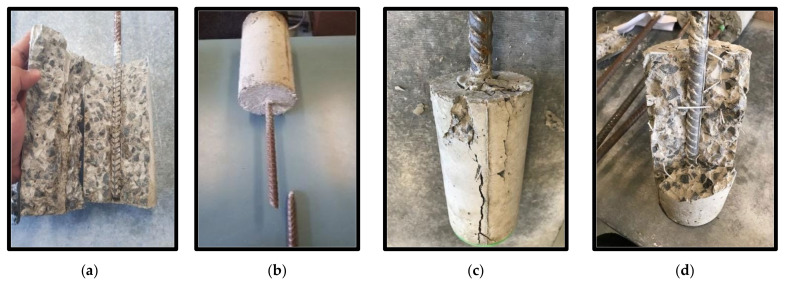
Failure mode of the tested samples against pull-out test (**a**) plain concrete (**b**) bar rupture, (**c**) Concrete splitting 16 mm and (**d**) Concrete splitting 20 mm.

Table 7 summarises different modes of failure as observed from testing the specimens with three fibre dosages (0 kg/m^3^, 4 kg/m^3^ and 6 kg/m^3^) and three steel bar diameters (12 mm, 16 mm and 20 mm). In Figure 16a, the plain concrete specimens showed splitting parallel to the axis of the bar in the concrete cylinders regardless of the bar diameter. This can be attributed to the brittle tensile failure of the concrete once the bar is subjected to pulling force because of the pushing out stress induced by the bonding between the steel bar threads and concrete. Interestingly, the specimens with 4 kg/m^3^ fibre dosage showed bar rupture (Figure 16b), when using 12 mm bar diameter, and concrete cracking (Figure 16c,d), when using 16 mm and 20 mm bar diameters. Specimens with 6 kg/m^3^ fibre dosage showed similar behaviour to the ones with 4 kg/m^3^ fibre dosage. The increase in the tensile capacity of the concrete increased the bonding strength between the steel bars and concrete, leading to bar rupture in the case of small bar diameter (12 mm) and massive concrete cracking in the case of 16 mm and 20 mm bar diameter. It was observed a number of PP fibres tying the cracked concrete, as seen in Figure 16d, as reinforcements against the brittle splitting failure as seen in Figure 16a with the plain concrete specimens.

#### 3.4.2. Pull-Out Load and Bonding Strength

**Figure 17:**
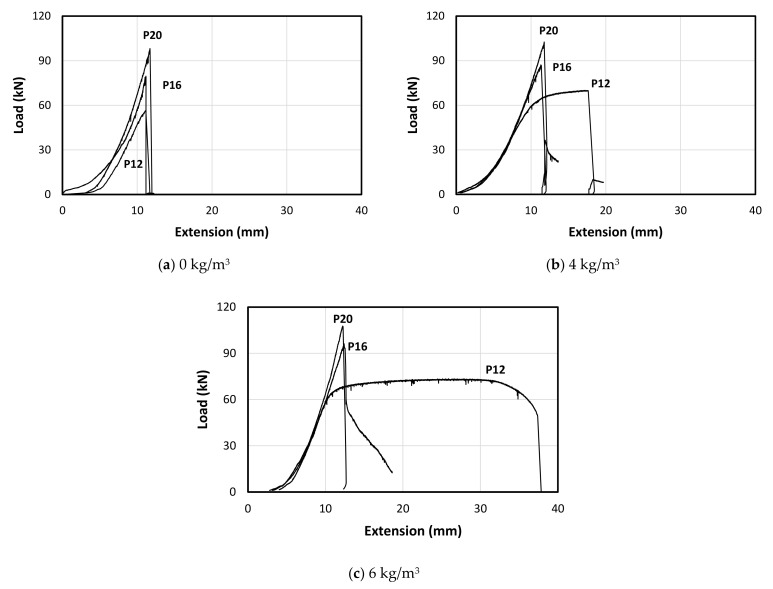
The typical pull-out load vs. the axial extension of the tested samples, (**a**) 0 kg/m^3^, (**b**) 4 kg/m^3^ and (**c**) 6 kg/m^3^.

Table 8 represents the test results of the pull-out load of the tested specimens, while Figure 16 shows the typical behaviour of the pull-out load versus the extension of the steel bars. In the case of plain concrete, it was observed that the increase in the bar diameter increases the pull-out failure load (see Figure 17a). This is due to the increase in bonding surface area between the bar and the concrete. The 12 mm bar showed a brief non-linear behaviour before the failure of the concrete which indicates reaching the yield strength of the steel bar (the minimum guaranteed yield stress is 500 MPa). However, the specimens with 16 mm and 20 mm showed sudden drop in the bonding resistance owing to the sudden split in the concrete as seen in Figure 16a. On the other hand, the 12 mm steel bar showed clear pseudo elastic-plastic behaviour after reaching the yielding stress (see Figure 17b). This is due the increase in the splitting tensile strength of the concrete which allows higher bonding capacity. However, the bar was pulled out after massive cracking at the outer surface of the concrete as typically seen in Figure 16c. Moreover, the 16 mm and 20 mm bar diameter showed similar behaviour to the ones with plain concrete with higher failure load (Figure 17b) due to the higher splitting tensile strength of the concrete. Adding 6 kg/m^3^ fibre dosage to the concrete specimens allowed the 12 mm steel bar to reach its ultimate strain limit and show complete elastic-plastic pseudo behaviour, as shown in Figure 17c. This led to the rupture of the steel bar while the embedded length was still intact with the concrete as seen in Figure 16b. However, this fibre addition was not enough to show the same behaviour for both 16 mm and 20 mm steel bars due to their higher cross-sectional area (178% and 278%, respectively) compared to the 12 mm steel bar. Thus, they showed similar behaviour to the plain concrete specimens and the ones with 4 kg/m^3^ fibre dosage (see Figure 17c).

**Figure 18:**
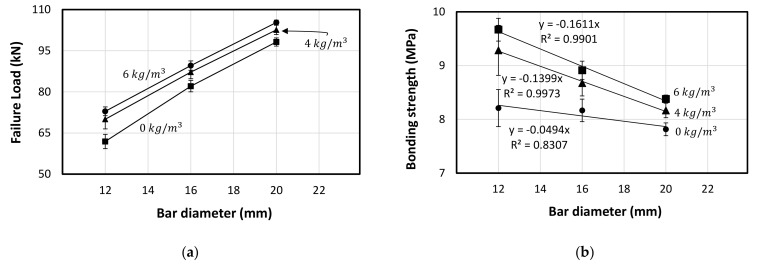
Bonding behaviour vs. bar diameter. (**a**) Failure load vs. Bar diameter. (**b**) Bond strength vs. Bar diameter.

On the other hand, Figure 18a showed the effect of the fibre addition on the final failure load where the increase in the fibre dosage increased the bonding strength between the concrete and the steel bar. Interestingly, this increase seems to be consistent linearly with the increase in the bar diameter. However, the bonding strength decreased with the increase in the bar diameter regardless of the fibre dosage addition (see Table 9). This is due to the fact that increasing the bar diameter increasing significantly the bar area ($\pi \times radius^{2}$) which causes significant different in the inertia between the concrete and the steel materials, even with increase in the bonding surface area which depends on the perimeter (2π×radius). Figure 18b represents the reduction in the bonding strength while increasing the bar diameter. However, the bonding strength increases with the increase in the fibre dosage due to the increase in the splitting tensile strength of the concrete. Moreover, it can be observed that the decrease rate in the bonding strength increases with the increase in the fibre dosage. This indicates the high impact of the fibrous concrete on the small bar diameter, while this impact decreases with larger bar diameter because of the inertia difference as mentioned. This finding is very important as it affects the required development length of the steel bars embedded in the fibrous concrete mentioned in the design codes. Thus, the next section suggested a simplified theoretical formula to capture this effect.

## 4. Theoretical Interpretation

**Figure 19:**
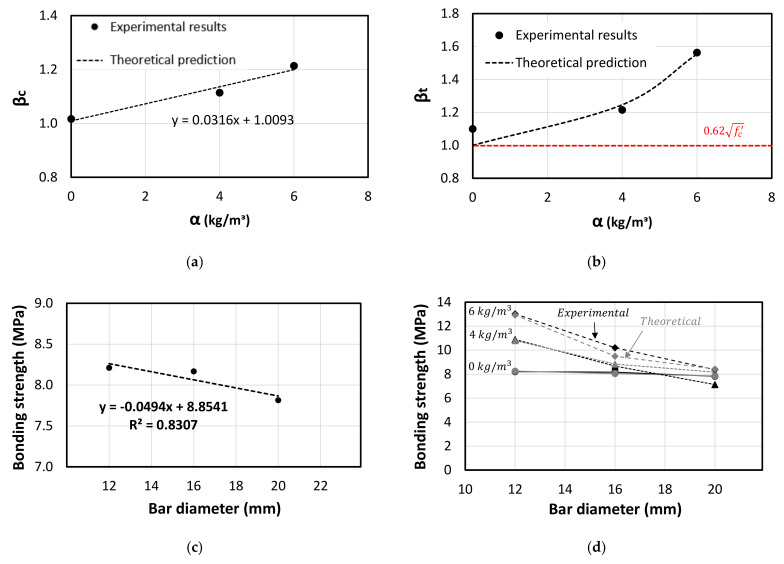
Predictions of mechanical properties of the concrete with fibre addition. (**a**) Compressive strength. (**b**) Splitting tensile strength. (**c**) Bonding strength vs. bar diameter (0 kg/m^3^). (**d**) Bond strength.

The addition of the fibres showed a clear enhancement on the tested mechanical properties including compression, tensile, flexure and bond strength. Therefore, this study proposed simplified theoretical interpretations to quantify the effect of the fibre addition on these mechanical properties compared to the plain concrete specimens. Accordingly, Figure 19a plot the strength improvement factor ($\beta_{c}$) for specimens under compression compared to the plain concrete specimens. Thus, Equation (5) represents the predicted compressive strength of the fibrous concrete with various fibre dosage. On the other hand, the strength improvement caused by the fibre addition is observed to be higher for the splitting tensile property compared to the compression one, as shown in Figure 19b. This is attributed to the fact that the function of the fibres is more on resisting and distributing the tensile and shear forces and delaying the crack propagation. Moreover, it can be observed that the tensile strength of the plain concrete specimens was close to the theoretical value ($0.62\sqrt{f_{c}^{\prime }}$) recommended by ACI-318 [49] for reinforced concrete structures. Thus, the theoretical predictions are based on this value ($0.62\sqrt{f_{c}^{\prime }}$).

On the other hand, the general formula of the bonding strength of the plain concrete (τ) is given in Equation (7), in which the τ is negatively affected by the bar diameter ($d_{b}$) evidenced by the linearly-decrease relationship as shown in Figure 19c. Therefore, in this study the bonding strength of the steel bars embedded in plain concrete can be calculated by Equation (7) (Figure 19c). This simplified equation is conditioned to the concrete strength and embedment length used in this study. To account for the increase in the fibre dosage on the bonding strength, it was noticed that the decrease rate in the bonding strength with the increase in the bar diameter is not constant and therefore a new reduction factor has been generated to account for the bar inertia and the amount of the fibre dosage as shown in Equation (8). Figure 19d shows the comparison between the experimental and theoretical bonding strength results considering various fibre dosages and different bar diameters. In summary, Table 10 reports the matching between the theoretical and experimental test results of the compression, splitting tensile and bond strength where the results of the proposed equations show good agreement with the experimental results. More comprehensive testing is required to verify the proposed equations.

These formulas need more verifying by considering a wide range of experimental data to validate the impact of fibres on these structural properties of concrete, while these formulas suggested in this study are only valid for this study. In the following equations, $\sigma_{c}$, $\sigma_{t}$ and $\sigma_{d}$ (MPa) are the theoretical concrete compressive, tensile and bond strength of the concrete (both with and without fibres), respectively. While $f_{c}^{\prime }$, $f_{t}$ and τ (MPa) are the experimental compressive, tensile and bond strength of the plain concrete. Moreover, F, $d_{b}$ and $h_{d}$ is the axial pull-out load (N), bar diameter (mm) and the embedment length (mm), respectively. Moreover, α is the percentage of fibre dosage in kg/m^3^:

(5) $$\sigma_{c}=\beta_{c}f_{c}^{\prime }=(1+\frac{3.2\times \alpha }{10^{2}})f_{c}^{\prime }$$

(6) $$\sigma_{t}=\beta_{t}f_{t}=\left(1+\frac{\alpha^{2}}{65}\right)f_{t}=\left(1+\frac{\alpha^{2}}{65}\right)0.62\sqrt{f_{c}^{\prime }}$$

(7) $$\tau =\frac{F}{\pi d_{b}h_{d}}=8.85-0.05d_{b}$$

(8) $$\sigma_{b}=\beta_{d}\tau =\left(1+\frac{800{\left(\alpha \right)}^{\frac{3}{2}}}{d_{b}{}^{4}}\right)\left[8.85-0.05d_{b}\right]$$

## 5. Microscopic Analysis

**Figure 20:**
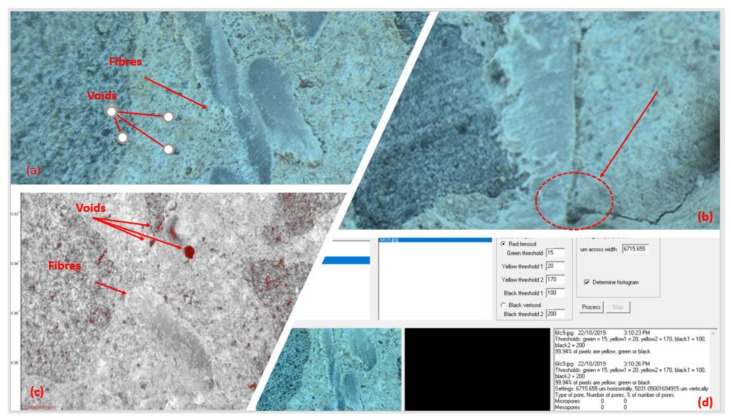
Stereo microscopic image showing the internal porosity and cracks (**a**) voids, (**b**) Control internal cracking, (**c**) image of voids and fibres and (**d**) TBitmap software used.

The fracture surface of the specimen with and without fibres was imaged using the TBitmap software. Moreover, the porosity analysis was conducted for specimens with various fibre dosages using the Gwyddion software. The stereomicroscope image in Figure 20 shows the ability of longitudinal macro polyfibres to control internal cracking resulting in enhancing the structural properties as mentioned in the previous sections. In this order, this will lead to increase the durability of concrete in general as the fibres are able to block the internal crack initiation and propagation [50].

Even though increasing the fibre dosage affects the total porosity, the effect of porosity on the compressive strength will not be based on the total porosity but rather on the pore size distribution. Based on Kay [51] method, the total porosity was classified based on the pore size distribution into three different sizes: (a) micro pores (<0.32 μm); (b) meso pores (0.2–30 μm); and (c) macro pores (>30 μm). Previous studies have indicated that the compressive strength depends upon the amount of macroporosity [52] and pore structure [53]. The pore structure of a porous material can be characterised by parameters such as pore size, pore conductivity, pore surface roughness, and pore volume fraction [54]. The size distribution of pores also affects the strength. Luping [55] found that a material with low porosity, but with more large pores, may have a lower strength than a material with a higher porosity and less large pores.

**Figure 21:**
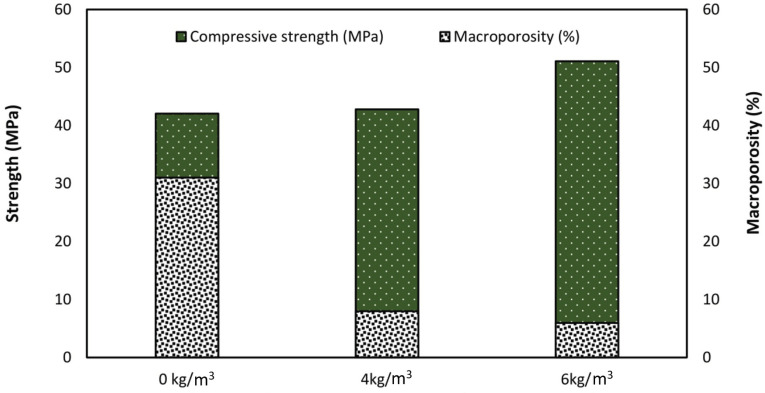
Compressive strength and macroporosity of concrete with different fibre dosages.

Figure 21 shows the relationship between the macroporosity and compressive strength attained with different dosage of fibres (0, 4 and 6 kg/m^3^) which agreed with the microscope observation. Increasing the amount of fibres decreased the macroporosity, A previous study conducted by Al-Harthi, Al-Amri [52] concluded that macroporosity affects the compressive strength of concrete, while the compressive strength is based on the amount of macroporosity, while another study by Price, Boyd [53] indicated that the pore structure affects the compressive strength. In this study the microporosity and mesoporosity did not have the same effect as the macroporosity.

## 6. Conclusions

This study investigated the effect of different amounts of macro polyfibre inclusion on the physical, mechanical, and micro-structural properties of concrete. Based on the results obtained by the wide range of testing conducted in this study, the following conclusions can be draw:

- Increasing the fibre dose from 4 kg/m^3^ to 6 kg/m^3^ slightly reduced the workability of the fresh concrete mix (up to 8.1%) due to the network structure formation in the concrete by the fibres which restrains the fresh concrete from flow.
- Macro polyfibres changed the mode of failure of the concrete cylinders under compression from longitudinal splitting to shear failure. Moreover, the inclusion of the 4 kg/m^3^ and 6 kg/m^3^ fibre dosages increased the compressive strength by 9.6% and 19.4% compared to the plain concrete, respectively. However, fibre addition has no significant effect on the modulus of elasticity value of the concrete due to the low volume of the added fibres by less than 0.7%
- Adding 4 kg/m^3^ and 6 kg/m^3^ fibre dosages to the concrete enhanced the splitting-tensile strength by 28.7% and 41.9%, respectively. This is due to the uniformly distribute of fibres which resist the internal tensile stresses as well as blocking the initiation of the cracks and reinforcing them once initiated against propagation.
- The fibre addition has no influence on the modulus of rupture of the concrete specimens due to the flexural loading mechanism which allows the fibre to function only after initiating the crack. However, the fibre addition contributed significantly to the flexural toughness of the tested beams where the beams with 6 kg/m^3^ fibre dosage attained 228% and 857% increase in the toughness compared to the beams with 4 kg/m^3^ and 0 kg/m^3^ fibre dosage, respectively. Moreover, the fibre addition changed the final failure from sudden and brittle to progressive failure.
- The fibrous concrete beams showed progressive crack mouth opening displacement (CMOD) more than 5 mm, while the plain concrete beam failure once recorded CMOD around 0.5 mm. It was observed that there is a relationship between the CMOD and the deflection indicating the stable behaviour of the fibrous concrete even after cracking.
- It was observed that increasing the bar diameter increased the pull-out force and however decreased the bonding strength as the latter negatively proportioned to the bar diameter. Moreover, all the steel bar diameter showed a similar mode of failure by splitting the concrete cylinder in a brittle manner. In contrast, adding macro poly fibres increased the bonding strength compared to the plain concrete and changed the mode of failure making the concrete cylinder failing in progressive way for the bars with 16 mm and 20 mm and interestingly a bar rupture in the 12 mm bar diameter.
- Theoretical interpretations on the test results were proposed thus they can predict the enhancement in the mechanical properties (compression and splitting tensile) of the concrete. New formulas were developed to predict the bonding strength of the concrete considering the fibre addition accounting for the fibre dosage and the steel bar diameter. These interpretations and formulas resulted in a good agreement with the experimental results.
- The stereo microscopic analysis revealed the ability of the macro polyfibres to control the internal cracks and reduce the inner pores by occupying and filling their locations. Furthermore, the pore size distribution influenced significantly the development of compressive strength more than the total porosity. More specifically, the macroporosity (>30 μm) has a direct correlation with the compressive strength, as opposed to the micropores and mesopores. Thus, the microporosity porosity in the concrete specimens decreased as the amount of fibres increased by 31%, 8% and 6%, respectively.

The outcome of this study will help to increase the implementation of the recycled plastic waste in the concrete mix design as a waste management pathway to minimise the environmental pollution. In addition, the result of this study will add to the existing knowledge of the functionality of these macro polyfibres in concrete structures and their ability to enhance structural performances. Therefore, it is recommended to utilise these macro polyfibres in full-scale concrete elements so that the holistic influence of these fibres in the concrete structures can be quantified.

## Figures and Tables

**Table 1:** Characteristics of macro polyfibre.

Cross section	1.2 × 0.5 mm^2^
Length	47 mm
Aspect ratio	94 (based on the shorter dimension)
Tensile strength	550 MPa
Density	910 kg/m^3^

**Table 2:** Tests conducted and specimen details.

Experiment Type	Fibre Dosage kg/m^3^	Number of Samples
Compression—AS-1012.9	0, 4 and 6	3 × 3 = 9
Splitting-tensile—AS-1012.9		5 × 3 = 15
Flexural—AS 1012.11		2 × 3 = 6
Bond slip (12, 16 and 20 mm)—ACI 440.03R-04		6 × 3 = 18
CMOD—BS EN 14651-2005		1 × 3 = 3

**Table 3:** Specification of displacement transducer.

Measuring capacity	0 mm to 5 mm
Sensitivity	0.001 strain/mm
Temperature range	0 to 40 °C
Gauge resistance	350 Ohm

**Table 4:** Compressive strength of concrete with different fibre content.

Fibre Dosage	Average $\sigma_{c}$ (MPa)	Standard Deviation (MPa)	Coefficient of Variation (%)
0 kg/m^3^	42.8	2.3	5.3
4 kg/m^3^	46.9	2.4	5.1
6 kg/m^3^	51.1	3.3	6.4

**Table 5:** Splitting tensile strength of concrete with different fibre dosage.

Fibre Dosage	Average $\sigma_{ts}$ (MPa)	Standard Deviation (MPa)	Coefficient of Variation (%)
0 kg/m^3^	4.46	0.57	12.8
4 kg/m^3^	4.93	0.36	6.7
6 kg/m^3^	6.34	0.17	2.7

**Table 6:** Flexural strength test results.

Fibre Dosage	Sample Number	Flexural Strength (MPa)	Average (MPa)	Toughness (kJ.m/m^2^)	Average (kJ.m/m^2^)
0 kg/m^3^	1	4.53	4.77 (0.59)	0.17	0.21 (0.03)
	2	5.01		0.24	
4 kg/m^3^	1	4.90	4.76 (0.34)	0.71	0.79 (0.08)
	2	4.62		0.87	
6 kg/m^3^	1	5.01	4.90 (0.28)	1.57	1.80 (0.23)
	2	4.79		2.03	

**Table 7:** Various mode of failure of the pull-out test.

Bar Diameter (mm)	Fibre Dosage (kg/m^3^)		
	0	4	6
12	Concrete splitting	Concrete Cracking	Bar Rupture
16	Concrete splitting	Concrete Cracking	Concrete Cracking
20	Concrete splitting	Concrete Cracking	Concrete Cracking

**Table 8:** Pull-out test results (kN) for the tested samples.

Bar Diameter (mm)	Fibre Dosage (kg/m^3^)					
	0		4		6	
	Average	SD	Average	SD	Average	SD
12	61.9	2.6	70.0	3.5	72.9	1.6
16	79.6	2.1	87.2	2.4	89.6	1.7
20	98.2	1.5	102.6	1.7	105.3	0.9

**Table 9:** Bonding strength results (MPa) for the tested samples.

Bar Diameter (mm)	Fibre Dosage (kg/m^3^)					
	0		4		6	
	Average	SD	Average	SD	Average	SD
12	8.21	0.34	9.28	0.46	9.67	0.21
16	8.17	0.21	8.67	0.24	8.91	0.17
20	7.81	0.12	8.16	0.14	8.38	0.07

**Table 10:** Comparison between theoretical and experimental results (MPa).

Fibre Dosage		0 kg/m^3^		4 kg/m^3^		6 kg/m^3^	
		Theoretical	Error (%)	Theoretical	Error (%)	Theoretical	Error (%)
Compression		43.23	+1	48.62	+4	51.31	+1
Splitting tensile		4.06	−9	5.06	+3	6.31	−1
Bonding	12 mm	8.25	+1	10.80	−1	12.93	−1
	16 mm	8.05	−2	8.84	+2	9.49	−7
	20 mm	7.85	+1	8.16	+14	8.43	+1

## Data Availability

Not applicable.

## References

[1] Apostolopoulos C.A., Demis S., Papadakis V.G. (2013). Chloride-induced corrosion of steel reinforcement—Mechanical performance and pit depth analysis. Constr. Build. Mater..

[2] Papakonstantinou K., Shinozuka M. (2013). Probabilistic model for steel corrosion in reinforced concrete structures of large dimensions considering crack effects. Eng. Struct..

[3] Daniel J.I.G.V., Galinat M.A. (2002). State-of-the-Art Report on Fiber Reinforced Concrete.

[4] Beglarigale A., Yazıcı H. (2015). Pull-out behavior of steel fiber embedded in flowable RPC and ordinary mortar. Constr. Build. Mater..

[5] Buratti N., Ferracuti B., Savoia M. (2013). Concrete crack reduction in tunnel linings by steel fibre-reinforced concretes. Constr. Build. Mater..

[6] Yuan C., Chen W., Pham T., Hao H. (2018). Bond behavior between basalt fibres reinforced polymer sheets and steel fibres reinforced concrete. Eng. Struct..

[7] Söylev T.A., Özturan T. (2014). Durability, physical and mechanical properties of fiber-reinforced concretes at low-volume fraction. Constr. Build. Mater..

[8] Tassew S., Lubell A. (2014). Mechanical properties of glass fiber reinforced ceramic concrete. Constr. Build. Mater..

[9] Sayyar M., Soroushian P., Sadiq M.M., Balachandra A., Lu J. (2013). Low-cost glass fiber composites with enhanced alkali resistance tailored towards concrete reinforcement. Constr. Build. Mater..

[10] Yin S., Tuladhar R., Shi F., Combe M., Collister T., Sivakugan N. (2015). Use of macro plastic fibres in concrete: A review. Constr. Build. Mater..

[11] Pujadas P., Blanco A., Cavalaro S., de la Fuente A., Aguado A. (2014). Fibre distribution in macro-plastic fibre rein-forced concrete slab-panels. Constr. Build. Mater..

[12] McMahon J.A., Birely A.C. (2018). Service performance of steel fiber reinforced concrete (SFRC) slabs. Eng. Struct..

[13] Shah S.P., Daniel J.I., Ahmad S.H., Arockiasamy M., Balaguru P., Ball C.G., Ball H.P., Batson G.B., Bentur A., Craig R.J. (1988). Measurement of properties of fiber reinforced concrete. ACI Mater. J..

[14] Kumar R., Naik T. Utilization of Post-Consumer Plastics in Sustainable Concrete: An Overview. Proceedings of the Fourth International Conference on Sustainable Construction Materials and Technologies.

[15] Kumar R., Goel P., Mathur R. Suitability of concrete reinforced with synthetic fiber for the construction of pavements. Proceedings of the 3rd International Conference on Sustainable Construction Materials and Technologies.

[16] Cominoli L., Plizzari G.A., Massinari P. (2007). Synthetic Fibre Reinforced Concrete for Precast Panels: Material Characteriza-tion and Experimental Study. Mater. Sci..

[17] Yin S., Tuladhar R., Collister T., Combe M., Sivakugan N., Deng Z. (2015). Post-cracking performance of recycled polypropylene fibre in concrete. Constr. Build. Mater..

[18] Ghosni N., Samali B., Vessalas K. Evaluation of structural behaviour of polypropylene fibre reinforced concrete beam under cyclic loading. Proceedings of the 23rd Australasian Conference on the Mechanics of Structures and Materials (ACMSM23).

[19] Vandewalle L. Postcracking behaviour of hybrid steel fiber reinforced concrete in Fracture Mechanics of Concrete and Concrete Structures—FraMCoS. Proceedings of the 6th International Conference.

[20] Clarke T. A cost-effective alternative to conventional concrete track slab design and construction. Proceedings of the AusRAIL PLUS 2016, Rail-Moving the Economy Forward.

[21] Altoubat S., Roesler J., Rieder K. (2008). Flexural capacity of synthetic fiber reinforced concrete slabs on ground based on beam toughness results. Proceedings of the 6th International RILEM Symposium on Fibre Reinforced Concretes.

[22] Pujadas P., Blanco A., Cavalaro S.H., de la Fuente A., Aguado A. Flat suspended slabs reinforced only with macro-synthetic fibres. Proceedings of the 9th RILEM International Symposium on Fiber Reinforced Concrete (BEFIB 2016).

[23] Altoubat S., Yazdanbakhsh A., Rieder K.-A. (2009). Shear Behavior of Macro-Synthetic Fiber-Reinforced Concrete Beams without Stirrups. ACI Mater. J..

[24] Navas F.O., Navarro-Gregori J., Herdocia G.L., Serna P., Cuenca E. (2018). An experimental study on the shear behaviour of reinforced concrete beams with macro-synthetic fibres. Constr. Build. Mater..

[25] Arslan G., Keskin R.S.O., Ozturk M. (2017). Shear behaviour of polypropylene fibre-reinforced-concrete beams without stirrups. Proc. Inst. Civ. Eng. Struct. Build..

[26] Yu P., Manalo A., Ferdous W., Abousnina R., Salih C., Heyer T., Schubel P. (2021). Investigation on the physical, mechanical and microstructural properties of epoxy polymer matrix with crumb rubber and short fibres for composite railway sleepers. Constr. Build. Mater..

[27] Standards Australia (1993). AS 1726, Geotechnical Site Investigations.

[28] Standards Australia (2009). AS 1141.11.1, Methods for Sampling and Testing Aggregates-Particle Size Distribution-Sieving Method.

[29] British Standard (2005). BSEN14651, Test Method for Metallic Fibre Concrete-Measuring the Flexural Tensile Strengths (Limit of Proportionali-ty (LOP), Residual) (+A1:2007).

[30] Standards Australia (2014). AS-1012.2 2014, Methods of Testing Concrete-Preparing Concrete Mixes in The Laboratory Standards Australia.

[31] Larsen I.L., Thorstensen R.T. (2020). The influence of steel fibres on compressive and tensile strength of ultra high performance concrete: A review. Constr. Build. Mater..

[32] Standards Australia (2014). AS-1012.9, Compressive Strength Tests Concrete, Mortar and Grout Specimens 2014.

[33] Standards Australia (2001). AS 1774.31.2, Refractories and Refractory Materials-Physical Test Methods, Modulus of Elasticity-Compression Method in (R2013) 2001.

[34] American Concrete Institute (2004). ACI 440.03R-04, F., Guide Test Methods for FRPs for Reinforcing or Strengthening Concrete Structures.

[35] Chen B., Liu J. (2005). Contribution of hybrid fibers on the properties of the high-strength lightweight concrete having good workability. Cem. Concr. Res..

[36] Abousnina R., Alsalmi H.I., Manalo A., Allister R.L., Alajarmeh O., Ferdous W., Jlassi K. (2021). Effect of Short Fibres in the Mechanical Properties of Geopolymer Mortar Containing Oil-Contaminated Sand. Polymers.

[37] Guerini V., Conforti A., Plizzari G., Kawashima S. (2018). Influence of Steel and Macro-Synthetic Fibers on Concrete Properties. Fibers.

[38] Standards Australia (2015). ASTM C39, Standard Test Method for Compressive Strength of Cylindrical Concrete Specimens.

[39] Abousnina R., Manalo A., Ferdous W., Lokuge W., Benabed B., Al-Jabri K.S. (2020). Characteristics, strength development and microstructure of cement mortar containing oil-contaminated sand. Constr. Build. Mater..

[40] Siddika A., Hajimohammadi A., Mamun A., Alyousef R., Ferdous W. (2021). Waste Glass in Cement and Geopolymer Concretes: A Review on Durability and Challenges. Polymers.

[41] Hasan M., Afroz M., Mahmud H. (2011). An experimental investigation on mechanical behavior of macro synthetic fiber reinforced concrete. Int. J. Civ. Environ. Eng..

[42] Li Z., Wang L., Wang X. (2004). Compressive and flexural properties of hemp fiber reinforced concrete. Fibers Polym..

[43] Song P., Hwang S., Sheu B. (2005). Strength properties of nylon- and polypropylene-fiber-reinforced concretes. Cem. Concr. Res..

[44] Lee S.-C., Oh J.-H., Cho J.-Y. (2015). Compressive Behavior of Fiber-Reinforced Concrete with End-Hooked Steel Fibers. Materials.

[45] King J. (2016). Behaviour of Concrete with Oil Contamination and Reinforced with Fibres.

[46] Chalioris C. (2013). Steel fibrous RC beams subjected to cyclic deformations under predominant shear. Eng. Struct..

[47] Choi S.-W., Choi J., Lee S.-C. (2019). Probabilistic analysis for strain-hardening behavior of high-performance fi-ber-reinforced concrete. Materials.

[48] Ding Y. (2011). Investigations into the relationship between deflection and crack mouth opening displacement of SFRC beam. Constr. Build. Mater..

[49] ACI (American Concrete Institute) (2014). Building Code Requirements for Structural Concrete ACI 318–14M.

[50] Khotbehsara M.M., Manalo A., Aravinthan T., Ferdous W., Benmokrane B., Nguyen K.T. (2021). Synergistic effects of hygrothermal conditions and solar ultraviolet radiation on the properties of structural particulate-filled epoxy polymer coatings. Constr. Build. Mater..

[51] Kay B. (2018). Soil Structure and Organic Carbon: A Review. Soil Process. Carbon Cycle.

[52] Al-Harthi A., Al-Amri R., Shehata W. (1999). The porosity and engineering properties of vesicular basalt in Saudi Arabia. Eng. Geol..

[53] Price R., Boyd P., Noel J., Martin R. (1994). Relation Between Static and Dynamic Rock Properties in Welded and Non-Welded Tuff.

[54] Corapcioglu M.Y. (1996). Advances in Porous Media.

[55] Luping T. (1986). A study of the quantitative relationship between strength and pore-size distribution of porous materials. Cem. Concr. Res..

